# Relationship between Iron Deficiency and Thyroid Function: A Systematic Review and Meta-Analysis

**DOI:** 10.3390/nu15224790

**Published:** 2023-11-15

**Authors:** Vincenzo Garofalo, Rosita A. Condorelli, Rossella Cannarella, Antonio Aversa, Aldo E. Calogero, Sandro La Vignera

**Affiliations:** 1 Department of Clinical and Experimental Medicine, University of Catania, 95124 Catania, Italy; vgarofalo985@gmail.com (V.G.); rossella.cannarella@phd.unict.it (R.C.); acaloger@unict.it (A.E.C.); sandrolavignera@unict.it (S.L.V.); 2 Cleveland Clinic Foundation, Glickman Urological & Kidney Institute, Cleveland, OH 44195, USA; 3 Department of Experimental and Clinical Medicine, University Magna Graecia of Catanzaro, 88100 Catanzaro, Italy; aversa@unicz.it

**Keywords:** iron deficiency, thyroid function, thyroid dysfunction, thyroid disease, hypothyroidism

## Abstract

**Objective:** Iron deficiency (ID) is the most prevalent nutritional deficiency worldwide. Low levels of serum ferritin (SF) could affect the thyroid gland and its functioning. The purpose of this systematic review and meta-analysis is to summarize the main currently available evidence and analyze data on the relationship between ID and thyroid function. **Methods:** This study included all articles evaluating the relationship between ID and thyroid function. Quality assessment was performed using Cambridge Quality Checklists. The search strategy included the following combination of Medical Subjects Headings terms and keywords: “iron deficiency”, “thyroid function”, “thyroid disease”, “thyroid dysfunction”, and “hypothyroidism”. A meta-analysis was performed to evaluate whether thyroid stimulating hormone (TSH), free thyroxine (FT4), and free triiodothyronine (FT3) levels differed between patients with ID and healthy controls without ID. For statistical comparison between cases and controls, the mean difference (MD) was calculated, and a subgroup analysis of pregnant and non-pregnant women was performed. Cochran’s Q testing and heterogeneity indices (*I*^2^) were used to assess statistical heterogeneity. Sensitivity analysis and publication bias analyses were also performed, both qualitatively and quantitatively. Finally, a meta-regression analysis was performed to evaluate the correlation between serum TSH or FT4 levels and SF in the study population. **Results:** Ten cross-sectional studies were identified and reviewed. Patients with ID showed TSH (MD: −0.24 mIU/L; 95% CI −0.41, −0.07; *I*^2^ = 100%, *p* = 0.005), FT4 (MD: −1.18 pmol/L; 95% CI −1.43, −0.94; *I*^2^ = 99%, *p* < 0.000001), and FT3 (MD: −0.22 pmol/L; 95% CI −0.32, −0.12; *I*^2^ = 99%, *p* < 0.00001) levels that were significantly lower. Subgroup analysis confirmed significantly lower TSH, FT4, and FT3 levels in pregnant women. Non-pregnant women showed significantly lower serum FT4 and FT3 levels but no difference in TSH values. Meta-regression analysis showed that serum TSH and FT4 levels were positively correlated with SF levels. Our systematic review of the literature found that ID significantly increases the prevalence of thyroid autoantibody (anti-thyroglobulin antibodies and anti-thyroid peroxidase antibodies) positivity both individually and collectively. **Conclusion:** Studies currently published in the literature indicate a possible relationship between ID, thyroid function, and autoimmunity, especially in some patient groups. Data analysis shows that thyroid hormone levels are lower in patients with ID and, in particular, in pregnant women. Further studies are needed to understand the role played by iron in thyroid metabolism.

## 1. Introduction

Iron is an essential nutrient required for various physiological functions. Iron deficiency (ID) is a widespread nutritional disorder worldwide, affecting about two billion people, mainly pregnant women and women of childbearing age [1,2]. ID is a condition in which the body lacks adequate amounts of iron. Iron is a key component of hemoglobin, a protein present in red blood cells that carries oxygen from the lungs to different tissues and organs in the body. It is also crucial for other enzymes and proteins involved in energy production and cell function. This common deficiency can lead to adverse effects on the thyroid gland, especially the function of the thyroid peroxidase enzyme [3,4]. Furthermore, anemia due to ID is a common comorbidity in patients with thyroid dysfunction, affecting nearly 5% of the population [5]. 

A growing body of evidence suggests that ID may play a significant role in the pathogenesis of thyroid dysfunction. Several studies have reported a high prevalence of ID in patients with thyroid diseases, particularly hypothyroidism and thyroid autoimmunity (TAI), which can impair the synthesis and function of thyroid hormones [6]. The production of thyroid hormones is negatively affected by ID, and their deficiency reduces the proliferation of erythrocyte precursors, both directly and through reduced secretion of erythropoietin by the kidneys [7,8]. Additionally, ID can affect the hypothalamic–pituitary–thyroid axis, leading to altered thyroid hormone levels and a decreased response to thyroid-stimulating hormone. Iron is also essential for the activity of thyroid peroxidase, an enzyme that catalyzes the iodination of tyrosine residues in thyroglobulin, a precursor protein for thyroid hormone synthesis [9]. Correlation between ID and hypothyroidism is likely due to impaired thyroperoxidase (TPO) hemoprotein biosynthesis, as shown in a rat study in which ID reduced TPO activity [4]. Furthermore, animal studies have shown that ID can interfere with thyroxine deiodinase activity by reducing the conversion of thyroxine (T4) to triiodothyronine (T3) and with the regulation of thyroid metabolism at the central level [10,11]. Moreover, the interaction between thyroid hormones and iron is bidirectional since, through the TRα receptor, TH directly stimulates erythropoiesis [12,13].

Despite growing interest in the relationship between ID and thyroid dysfunction, scientific evidence is still inconclusive. Several studies have been conducted to evaluate the association between iron status and thyroid function, but the results are conflicting. While some studies suggest that ID is associated with an increased risk of thyroid dysfunction, others have found no significant association.

A recent systematic review and meta-analysis show that the prevalence of overt and subclinical hypothyroidism is higher in pregnant women and women of childbearing age with ID who had higher thyroid-stimulating hormone (TSH) values and reduced free thyroxine (FT4) values, with a possible increase in the risk of autoantibody positivity [14]. Therefore, a comprehensive and up-to-date systematic review of the literature and meta-analysis is needed to provide a better understanding of the relationship between ID and thyroid dysfunction in the general population. This study aims to summarize and critically evaluate the available evidence on the association between iron status and thyroid function in the general adult population, including the mechanisms underlying this association, the prevalence of ID in patients with thyroid diseases, and the impact of iron supplementation on thyroid function. In addition, this study aims to analyze currently available data on the impact of serum ferritin values on thyroid hormones.

## 2. Methods

### 2.1. Search Strategy

The Meta-Analysis and Systematic Reviews of Observational Studies (MOOSE) guidelines [15] (Supplementary Table S1) and the Preferred Reporting Items for Systematic Review and Meta-Analysis Protocols (PRISMA-P) [16] (Supplementary Table S2) were employed to carry out our systematic review. A systematic search was performed from April 2023 to July 2023, across the Pubmed and Scopus databases from the earliest available through July 2023, using Medical Subjects Headings (MeSH) indexes and keyword searches. The string used included the entry term “iron deficiency*”, which was searched in combination (AND) with the terms “thyroid function” OR “thyroid dysfunction” OR “thyroid disease” OR “hypothyroidism”. The “LIMIT-TO (DOCTYPE, “ar”)” query was used to limit the retrieval of original studies only. The same combination of terms was used for all databases. Abstracts of the retrieved articles were independently screened by two researchers in duplicate (V.G. and R.A.C.). Disagreements were resolved by a third person (A.E.C.).

### 2.2. Selection Criteria

This systematic review included all published articles evaluating the relationship between ID and thyroid function. All eligible studies were selected following the PECOS (population, exposure, comparison/comparator, outcomes, study design) [17] model (Table 1). Specifically, all the studies reporting information on thyroid function in patients exposed to ID, consisting of a decrease in extracellular iron with a lower-than-normal serum ferritin (SF), were included. Only original articles in English language reporting complete data of clinical relevance to the present review were included in the analysis. Duplicates have been carefully checked and removed.

### 2.3. Data Extraction

We collected information on the first author, year of publication, study design, age, type of population (gender, pregnant or non-pregnant), diagnostic criteria for ID and SF levels, and information on primary outcomes (TSH, FT4, FT3, TgAb, and TPOAb). For analysis, TSH values were converted to mIU/L and FT4 and FT3 in pmol/L, if provided in different units of measure, according to the conversion tables. For each parameter, the number of patients/controls, mean value and standard deviation (SD), median value and interquartile range (IQR) range, or median value and minimum and maximum values were reported, depending on how the authors reported the data. For studies that express data as median and IQR or as median and minimum and maximum, the formula by Wan and colleagues [18] was used to estimate the mean and SD. The data were independently extracted by V.G. 

### 2.4. Quality Assessment

The quality of evidence (QoE) of the studies was evaluated by VG, by using the Cambridge Quality Checklists [19], which consists of three checklists to identify high-quality studies of (1) correlates, (2) risk factors, and (3) causal risk factors. The correlates checklist evaluates the appropriateness of the sample size and the quality of the outcome measurements. The risk factor checklist assigns high-quality scores to those studies with appropriate time-ordered data. Finally, the causal risk factors checklist assesses the type of study design. To draw confident conclusions about correlates, risk factors, and causal risk factors, all three checklist scores must score high.

As no randomized control trials were included, no further evaluation was needed.

### 2.5. Statistical Analysis

Quantitative data analysis was performed using Comprehensive Meta-Analysis Software (Version 3) (Biostat Inc., Englewood, NJ, USA) and RevMan software v. 5.4 (Cochrane Collaboration, Oxford, UK). The mean difference (MD) was calculated for statistical comparison of cases and controls, including subgroup analysis between pregnant and non-pregnant women. Cochran’s Q testing and heterogeneity indices (*I*^2^) were used to assess statistical heterogeneity. In particular, if *I*^2^ was less than or equal to 50%, the variation of the studies was considered homogenous, and the fixed effect model was adopted to calculate the pooled effect size. However, if *I*^2^ was greater than 50%, there was significant heterogeneity between studies, and the random effect model was used. Sensitivity analysis and analysis of publication bias were also performed. Publication bias was qualitatively analyzed by the asymmetry of the funnel plot, which suggested some missing studies on one side of the graph. For the quantitative analysis of publication bias, we used Egger’s intercept test, which evaluated the statistical significance of publication bias. We also performed a meta-regression analysis to investigate the correlation between serum TSH of FT4 levels and SF in the study population. Statistical significance was accepted for *p*-values less than or equal to 0.05.

## 3. Results

**Figure 1:**
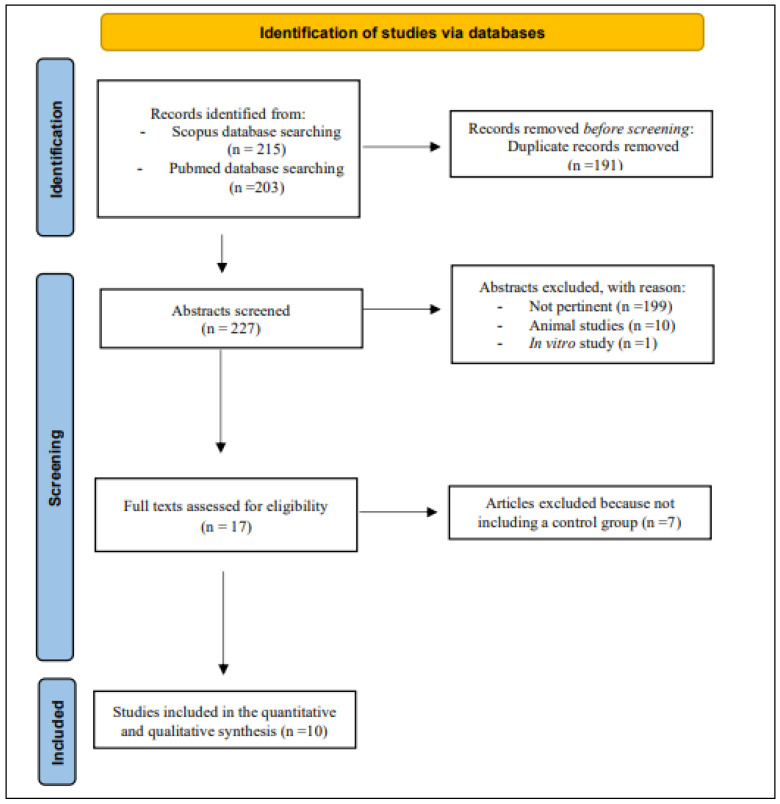
The PRISMA diagram for Databases and Registers [30].

The search strategy mentioned above identified a total of 418 records. After 191 duplicates were excluded, the remaining 227 articles were considered potentially relevant for this review. After reading the abstracts, 199 articles were excluded because they were not pertinent. Ten studies were conducted on animals, and one was an *in vitro* study. The full text of the remaining seventeen articles were downloaded and read carefully: seven of these were excluded because they did not include a control group or because data were insufficient. In conclusion, ten studies were considered for this systematic review (Figure 1).

The main characteristics of the included studies are reported in Table 2. Nine of them evaluated the association between ID and thyroid function in pregnant women or women of childbearing age [20,21,22,23,24,25,26,27,28], and only one study was conducted on the general population [29]. Among the included studies, they all evaluated different parameters regarding thyroid function. All studies [20,21,22,23,24,25,26,27,28,29] measured blood levels of TSH and FT4 to evaluate thyroid function, and four of them also evaluated serum FT3 levels [20,21,25,29]. Six studies [21,22,23,24,26,27] evaluated the positivity of TPOAb in the blood of patients, and four of these [21,22,23,24] evaluated the TgAb to determine the possible presence of autoimmune thyroiditis. For the diagnosis of ID, four studies [20,22,25,26] used ferritin values < 20 ng/dL, four studies [21,23,27,29] used ferritin values < 15 ng/dL, and two studies [24,28] values < 12 ng/dL. All studies used electrochemiluminescence immunoassay to measure blood levels of TSH, FT4, FT3, TPOAb, TgAb, and SF. The reference ranges of thyroid parameters are reported in Table 2.

### 3.1. Quality of Evidence of Included Studies

The QoE of ten studies included revealed that, out of a total score of fifteen, five studies scored an eleven, four studies scored a ten, and only one study scored a nine (Table 3), supporting the high quality of the majority of the studies.

### 3.2. Results of the Quantitative Synthesis

#### 3.2.1. TSH

**Figure 2:**
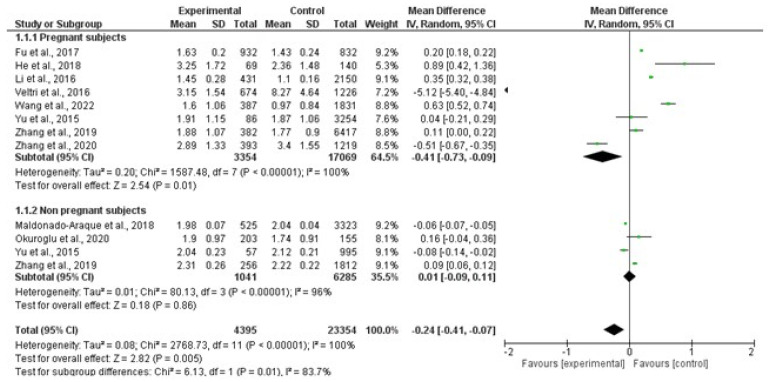
Forest plot of mean difference in serum TSH levels between patients and controls and subgroup analysis in pregnant and non-pregnant women. Green boxes estimate the study results, and give a representation of the size of the studies. Horizontal lines represent the 95% confidence intervals of the study results, with each end of the line representing the boundaries of the confidence interval. The black diamond represents the point estimate and confidence intervals of the combined studies. The left arrow indicates a study whose estimate point and confidence interval are lower than −2. Fu et al., 2017 [25]; He et al., 2018 [24]; Li et al., 2016 [26]; Veltri et al., 2016 [27]; Wang et al., 2022 [20]; Yu et al., 2015 [28]; Zhang et al., 2019 [23]; Zhang et al., 2020 [22]; Maldonado-Araque et al., 2015 [29]; Okuroglu et al., 2020 [21].

Ten studies, including 4395 patients and 23,354 controls, assessed serum TSH levels. Our analysis showed that, overall, TSH was significantly lower in patients than in controls, although interstudy heterogeneity was found (MD: −0.24 mIU/L; 95% CI −0.41, −0.07; *I*^2^ = 100%, *p* = 0.005). Subgroup analysis revealed significantly lower TSH values in the pregnant population (MD: −0.41 mIU/L; 95% CI −0.73, −0.09; *I*^2^ = 100%, *p* = 0.01), while they were non-significantly different in non-pregnant subjects (MD: 0.01 mIU/L; 95% CI −0.09, 0.11; *I*^2^ = 96%, *p* = 0.86) (Figure 2). 

**Scheme 1:**
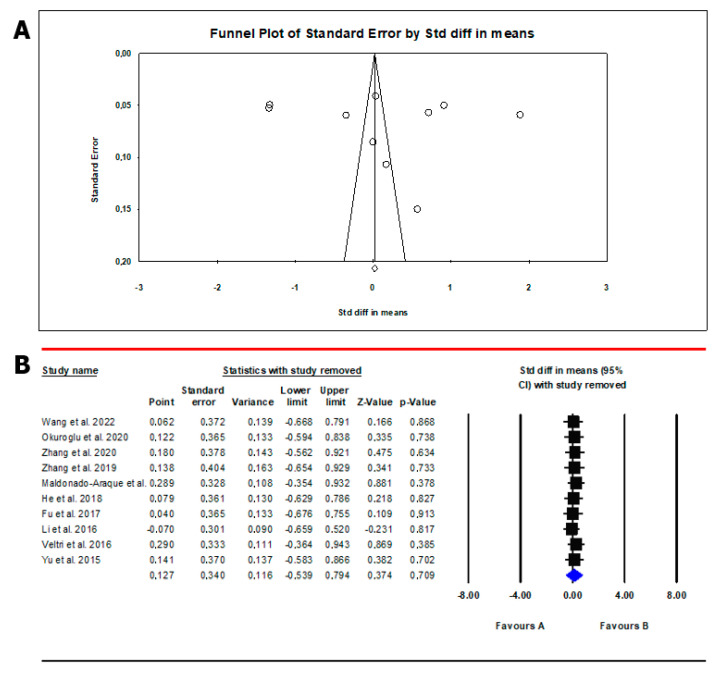
Funnel plot (**A**) and sensitivity analysis (**B**) of the mean difference in serum TSH values in patients and controls. In Panel (**A**), circles indicate the standard error plotted by the standard difference in means of each study. Rhombus indicates a standard difference in means of zero. In Panel (**B**), black boxes estimate the study results, and give a representation of the size of the studies. horizontal lines represent the 95% confidence intervals of the study results, with each end of the line representing the boundaries of the confidence interval. The blue diamond represents the point estimate and confidence intervals of the combined studies. Fu et al., 2017 [25]; He et al., 2018 [24]; Li et al., 2016 [26]; Veltri et al., 2016 [27]; Wang et al., 2022 [20]; Yu et al., 2015 [28]; Zhang et al., 2019 [23]; Zhang et al., 2020 [22]; Maldonado-Araque et al., 2015 [29]; Okuroglu et al., 2020 [21].

**Scheme 2:**
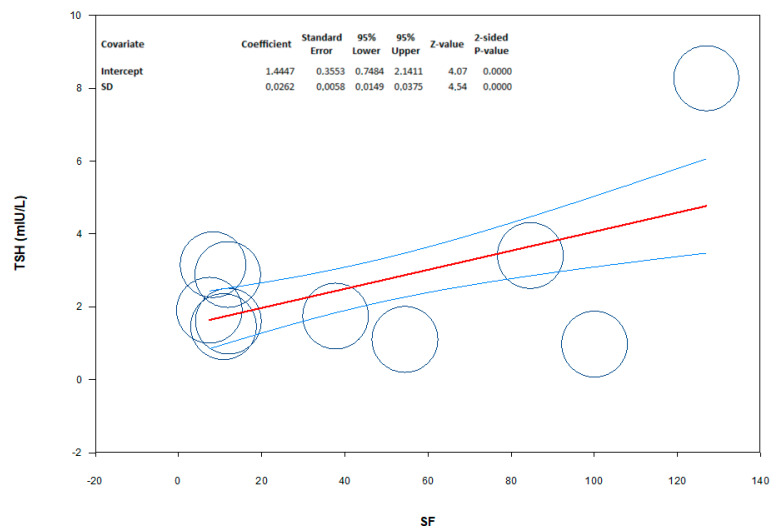
Meta-regression showing the influence of serum ferritin (SF) levels (moderator) on the mean difference of serum TSH levels in patients from the study population. The size of the circles indicates the size of the study. The red line represents linear predictions for mean difference of TSH as a function of the mean absolute increase in SF. The curved lines indicate the 95% confidence interval lines around the regression line.

The analysis showed the absence of publication bias as inferred by Egger’s test (intercept 8.12, 95% CI −37.7; 53.9; *p* = 0.69) and symmetry of the funnel plots (Scheme 1, panel A). However, all studies were found to be sensitive enough to change the conclusion that serum TSH levels are lower in patients than in controls (Scheme 1, panel B). Meta-regression analysis showed that serum TSH levels correlated positively with SF. In this analysis, the magnitude of mean serum levels in patients and controls increased as a function of their SF values (Scheme 2).

#### 3.2.2. Free Thyroxin

**Figure 3:**
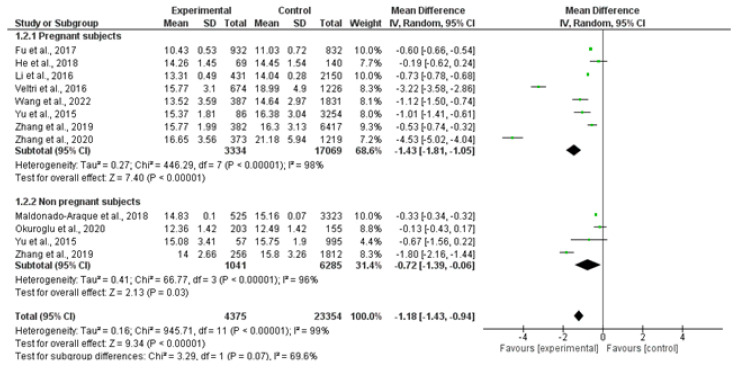
Forest plot of mean difference in serum free thyroxin levels between patients and controls and subgroup analysis in pregnant and non-pregnant women. Green boxes estimate the study results, and give a representation of the size of the studies. Horizontal lines represent the 95% confidence intervals of the study results, with each end of the line representing the boundaries of the confidence interval. The black diamond represents the point estimate and confidence intervals of the combined studies. Fu et al., 2017 [25]; He et al., 2018 [24]; Li et al., 2016 [26]; Veltri et al., 2016 [27]; Wang et al., 2022 [20]; Yu et al., 2015 [28]; Zhang et al., 2019 [23]; Zhang et al., 2020 [22]; Maldonado-Araque et al., 2015 [29]; Okuroglu et al., 2020 [21].

Ten studies, including 4395 patients and 23,354 controls, evaluated serum FT4 levels. FT4 was significantly lower in patients than in controls, despite interstudy heterogeneity (MD: −1.18 pmol/L; 95% CI −1.43, −0.94; *I*^2^ = 99%, *p* < 0.000001). By subgroup analysis, FT4 was lower in both pregnant women (MD: −1.43 pmol/L; 95% CI −1.81, −1.05; *I*^2^ = 98%, *p* < 0.000001) and non-pregnant women (MD: −0.72 pmol/L; 95% CI −1.39, 0.06; *I*^2^ = 96%, *p* = 0.03) (Figure 3). 

**Scheme 3:**
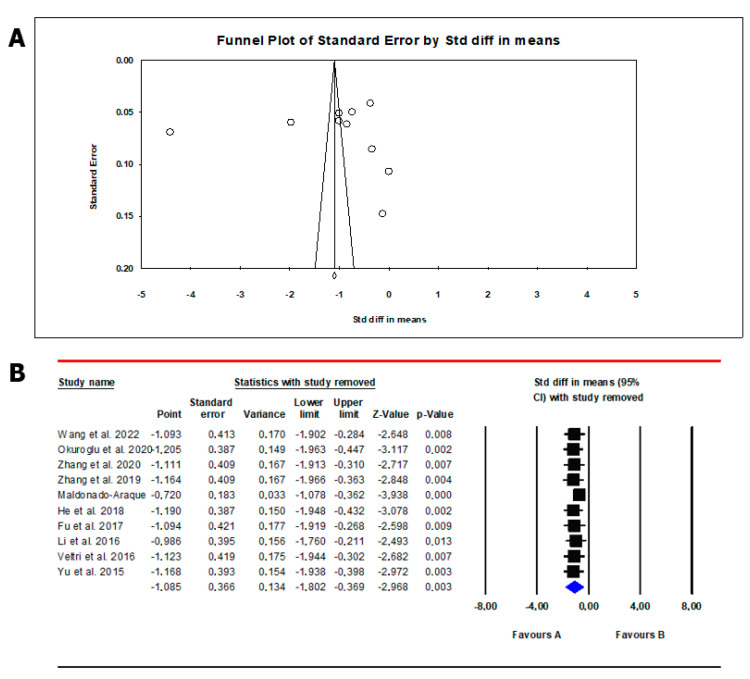
Funnel plot (**A**) and sensitivity analysis (**B**) of mean difference in serum free thyroxin (FT4) levels in patients and controls. In Panel (**A**), circles indicate the standard error plotted by the standard difference in means of each study. Rhombus indicates a standard difference in means of zero. In Panel (**B**), black boxes estimate the study results, and give a representation of the size of the studies. horizontal lines represent the 95% confidence intervals of the study results, with each end of the line representing the boundaries of the confidence interval. The blue diamond represents the point estimate and confidence intervals of the combined studies. Fu et al., 2017 [25]; He et al., 2018 [24]; Li et al., 2016 [26]; Veltri et al., 2016 [27]; Wang et al., 2022 [20]; Yu et al., 2015 [28]; Zhang et al., 2019 [23]; Zhang et al., 2020 [22]; Maldonado-Araque et al., 2015 [29]; Okuroglu et al., 2020 [21].

**Scheme 4:**
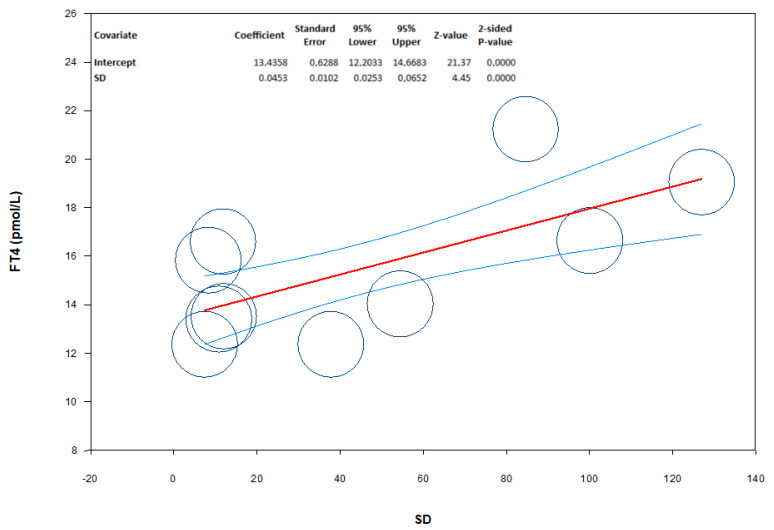
Meta-regression showing the influence of serum ferritin (SF) levels (moderator) on the mean difference in serum free thyroxin (FT4) levels among patients in the study population. The size of the circles indicates the sample size of the study. The red line represents linear predictions for the mean difference in FT4 as a function of the mean absolute increase in SF. The curved lines indicate the 95% confidence interval lines around the regression line.

The analysis showed the absence of publication bias as inferred by Egger’s test (intercept −5.35, 95% CI −52.8; 42.1; *p* = 0.80) and symmetry of the funnel plots (Scheme 3, panel A). No study was sensitive enough to alter the results (Scheme 3, panel B). In meta-regression analysis, we found a positive correlation between serum FT4 levels and SF levels in the study population. Indeed, the magnitude of mean serum FT4 levels in patients and controls increased as a function of their SF levels (Scheme 4).

#### 3.2.3. Free Triiodothyronine

**Figure 4:**
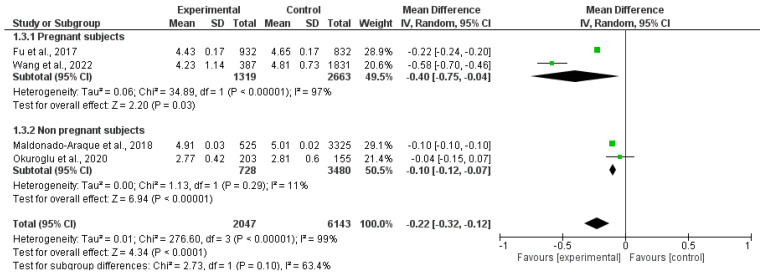
Forest plot of the mean difference in serum free triiodothyronine levels between patients and controls and subgroup analysis in pregnant and non-pregnant women. Green boxes estimate the study results, and give a representation of the size of the studies. Horizontal lines represent the 95% confidence intervals of the study results, with each end of the line representing the boundaries of the confidence interval. The black diamond represents the point estimate and confidence intervals of the combined studies. Fu et al., 2017 [25]; Wang et al., 2022 [20]; Maldonado-Araque et al., 2015 [29]; Okuroglu et al., 2020 [21].

Four studies, including 2047 patients and 6143 controls, evaluated serum FT3 levels. FT3 was significantly lower in patients than in controls, despite the presence of interstudy heterogeneity (MD: −0.22 pmol/L; 95% CI −0.32, −0.12; *I*^2^ = 99%, *p* < 0.00001). Its levels were lower in both pregnant (MD: −0.40 pmol/L; 95% CI −0.75, −0.04; *I*^2^ = 97%, *p* = 0.03) and non-pregnant women (MD: −0.10 pmol/L; 95% CI −0.12, −0.07; *I*^2^ = 11%, *p* < 0.00001) (Figure 4). 

**Scheme 5:**
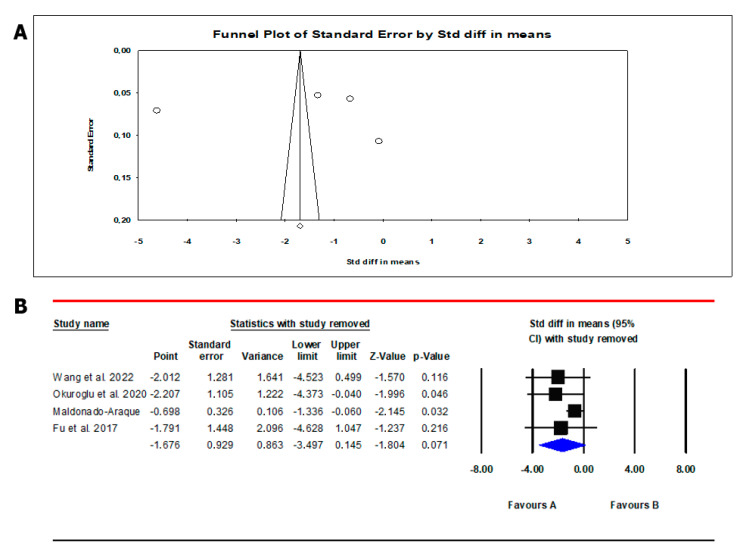
Funnel plot (**A**) and sensitivity analysis (**B**) of the mean difference in serum free triiodothyronine (FT3) levels in patients and controls. In Panel (**A**), circles indicate the standard error plotted by the standard difference in means of each study. Rhombus indicates a standard difference in means of zero. In Panel (**B**), black boxes estimate the study results, and give a representation of the size of the studies. horizontal lines represent the 95% confidence intervals of the study results, with each end of the line representing the boundaries of the confidence interval. The blue diamond represents the point estimate and confidence intervals of the combined studies. Fu et al., 2017 [25]; Wang et al., 2022 [20]; Maldonado-Araque et al., 2015 [29]; Okuroglu et al., 2020 [21].

The analysis showed the absence of publication bias as inferred by Egger’s test (intercept −6.9, 95% CI −312.2; 298.3; *p* = 0.93) and symmetry of the funnel plots (Scheme 5, panel A). Three studies were found to be sensitive enough to alter the conclusion that FT3 is lower in patients than in controls [20,21,25] (Scheme 5, panel B). 

### 3.3. Results of the Qualitative Synthesis

All studies showed a significant reduction in blood FT4 levels in patients with ID, and only two studies [20,29] showed a significant reduction in plasma FT3 levels. Regarding TSH levels, seven studies [20,21,22,24,25,26,27] reported a direct relationship with the severity of ID; ID patients in these studies had higher values than the control group without ID. However, three studies did not show significant differences in TSH levels between patients with and without ID [23,28,29]. This may be due to the different populations included across the studies. Indeed, some included non-pregnant women [23,28] and others included the general population [29]. 

Regarding autoimmunity, in patients with ID, an increase in TPOAb positivity prevalence was reported in four studies [21,23,26,27], while TgAb positivity prevalence was reported in two studies [21,22]. Okuroglu and colleagues reported an increase in the positivity prevalence of both autoantibodies in non-pregnant women of childbearing age [21]. Zhang Y. and collaborators are the only ones to report that ID is a risk factor for TgAb positivity and not for TPOAb during the second trimester of pregnancy [22]. However, in another study [23], the prevalence of isolated TPOAb positivity in the ID group was significantly higher than the control group in non-pregnant women, while the prevalence of isolated TgAb positivity was similar between the ID group and the control group, both in pregnant and non-pregnant women. The difference could be due to the presence of non-pregnant women in the second study. Li and colleagues, instead, showed greater positivity prevalence for TPOAb in women with ID in early pregnancy than in the control group [26]. Only one study [24] found no differences in TPOAb and TgAb levels in relation to SF levels.

## 4. Discussion

ID is the most prevalent nutritional deficiency worldwide. The most affected populations are pregnant women and women of childbearing age. The World Health Organization reports that ID is the leading cause of anemia in pregnant women (38.2%) and women of reproductive age (29.4%) [31]. Several studies have shown that ID has negative effects on thyroid function. Iron is an essential trace element for the biosynthesis and function of thyroid hormones. In the body, iron is mainly contained in hemoglobin and myoglobin, but a small part is present in various cytochromes and other hemoproteins, including thyroperoxidase (TPO), where it represents the central atom of the prosthetic groups in their active site. Expressed inside thyrocytes, TPO is the key enzyme in thyroid hormone synthesis, catalyzing the H_2_O_2_-dependent oxidative reaction of iodide ions to elemental iodine which, in turn, will be incorporated into thyroglobulin to produce thyroid hormones [32]. 

The most important clinical indicator of ID is SF, which reflects the body’s iron stores. Restoration of adequate SF concentrations promotes the recovery of normal thyroid function in ID girls [33]. Furthermore, in patients with ID and subclinical hypothyroidism, combined treatment with levothyroxine and iron supplements demonstrated better effects than either factor alone [34,35]. 

Most studies in the literature related to ID and thyroid function have been conducted on pregnant women and women of childbearing age, while only one study has been carried out on large samples of the general adult population [29]. Subclinical hypothyroidism ranges from 4% to 17% during pregnancy, and thyroid hormones play a crucial role in brain development, especially during the first months of intrauterine life [36]. Impaired thyroid function and TAI during pregnancy are associated with gestational diabetes mellitus, blood hypertension, miscarriage, fetal dysplasia, and neurological deficits in children [37]. Pregnant women are more vulnerable to ID as their needs increase due to the expansion of the erythrocyte mass and growth of the fetus and placenta [38]. Zimmermann and colleagues reported that an insufficient maternal iron reserve predicts lower blood total thyroxin levels and higher TSH levels during pregnancy [39]. 

In this systematic review and meta-analysis, we collected studies regarding the correlation between ID and thyroid function. A careful literature review collected cross-sectional studies comparing SF levels and thyroid function markers, specifically, TSH, FT4, FT3, TPOAb, and TgAb, in the general population of adults, pregnant women, or women of childbearing age.

Wang and colleagues, in their study of Chinese pregnant women with ID or ID anemia, observed that serum FT3 and FT4 values had a significant downward trend in the ID and ID anemia groups, with lower levels in the second group compared with the control group. In addition, significantly higher TSH values have been reported in pregnant women with ID or ID anemia. Finally, compared with the control group, the rate of hypothyroidism was higher in both these groups of patients. Using canonical correlation analysis, SF and hemoglobin values were positively correlated with FT3 and FT4 and negatively correlated with TSH, thus providing further evidence of the correlation between iron status and thyroid function. This was further validated by ROC curve analysis using selected variables from Lasso’s model to predict the level of FT4, where the accuracy was good [20]. 

Another study of Chinese women in the first trimester of pregnancy shows that the serum TSH level in women with SF < 20 ng/dL was significantly higher than in the SF 20–100 ng/dL and SF > 100 ng/dL groups. Conversely, the blood FT4 level in women with SF < 20 ng/dL is significantly lower than in groups with SF > 20 ng/dL. Furthermore, the relationship between urinary iodine concentration (UIC) with iron status and thyroid function was evaluated. Observations showed a positive and significant association between UIC and SF and between UIC and FT3 when UIC is less than 249 µg/L. In addition, observations showed a negative and significant association between UIC and SF and between UIC and FT3 levels when UIC is greater than 250 µg/L. These data indicate that more than adequate iodine nutritional status is associated with reduced SF and FT3 concentrations [25]. These results are in line with other studies where UIC correlates positively with serum TSH during pregnancy [40,41].

In contrast to the two previous studies, Yu and colleagues reported that both pregnant and non-pregnant women with ID had an increased risk of isolated hypothyroxinemia, with no significant differences in serum TSH levels [28]. The difference in results may be explained by the different iodine status, the presence of non-pregnant women in the study by Yu and colleagues, and the use of different cut-offs to define ID. Another explanation could be the lack of TAI assessment in these studies [20,25,28].

A study on a large sample of subjects without thyroid diseases, representative of the Spanish adult population, shows that subjects with SF levels < 30 mL/dL were more likely to have low FT4 and FT3 levels than subjects with SF ≥ 30 mg/dL, confirming a possible association between ID and hypothyroxinemia. However, mean TSH concentrations did not show significant changes [29]. 

Okuroglu and coworkers, in their study, support the association between ID and TAI. In the ID group, positivity for TPOAb plus TgAb was significantly higher than in the control group [21]. Zhang and colleagues found lower FT4 levels in pregnant and non-ID Chinese women than in the control group, but the median TSH levels were similar in both the ID and control groups. Regarding TAI, the prevalence of isolated TPOAb positivity was higher in the ID group than in women without ID. After adjusting for confounding factors in multivariable logistic regression, ID remained associated with isolated TPOAb positivity in both pregnant and non-pregnant women. However, ID was not associated with isolated TgAb positivity [23]. These results are in line with two previous studies in which the prevalence of positivity for TPOAb was higher in women with ID during early pregnancy and in which SF levels were directly proportional to FT4 levels and inversely proportional to TSH levels [26,27].

The mechanism by which ID is associated with TPOAb but not TgAb positivity remains unclear. It has been hypothesized that ID may produce post-translational changes in TPO, leading to the generation of new epitopes or the exposure of previously hidden epitopes and thus increasing the immunogenicity of TPO. Another hypothesis is that the decrease in TPO activity resulting from ID causes an increase in TAI [27].

Zhang et al. focused on the association of TAI and ID during the second trimester of pregnancy in Chinese women. Unlike other studies, they were the first to report that ID was a risk factor only for increased TgAb levels and not for TPOAb [22]. As previously mentioned, ID reduces the activity of heme-containing enzymes including myeloperoxidase (MPO). Women with ID may exhibit increased MPOAb which, in turn, may cross-react with AbTPO and, consequently, lead to increased thyroid autoantibody positivity [42]. At present, the mechanisms by which SF deficiency increases TgAb levels is not known. It is hypothesized that following SF deficiency, the decrease in FT4 results in a reduction of feedback inhibition on TSH, thereby increasing its levels. Since TSH promotes TG mRNA and TG antigen expression, this may lead to increased TgAb production [43]. 

Only one study [24] has excluded a correlation between TAI and ID in second-trimester pregnant women with normal urinary iodine levels, thus emphasizing an important role of this micronutrient in the pathogenesis of TAI [44].

Analysis of data on TSH and free thyroid hormones showed that their serum levels were significantly lower in ID patients than in controls, with lower values in pregnant women than in non-pregnant women. The behavior of TSH is curious: it goes in the same direction as FT4. This could be explained by the heterogeneity of the examined population, as serum TSH levels were lower in pregnant women than in the general population. However, the FT4 values should be more reliable than the TSH levels since the significance of the latter is lost in the sensitivity analysis. Meta-regression analysis shows that mean serum TSH and FT4 levels in patients and controls increase as a function of SF values, indicating a possible relationship between iron status and thyroid function. 

ID may also be associated with an increased risk of developing TAI, particularly in pregnant women, and should be included in the screening of women planning to have a child. However, most of the studies considered do not take into account iodine status, and some of them are conducted in areas with mild iodine deficiency. Also, other parameters should be considered. First, the role of inflammation on SF, particularly during pregnancy, as well as the role of other hormones that might affect thyroid function and iron status. Inflammation hinders the interpretation of iron biomarkers, particularly serum ferritin and hepcidin. In fact, several pro-inflammatory cytokines increase the synthesis of ferritin and hepcidin with a consequent increase in iron trapping within cells. In case of acute inflammation, serum ferritin levels of 50 ng/mL or greater may indicate ID. Therefore, inflammation reduces the predictive values of ferritin and hepcidin in the case of ID [45]. Recent studies have shown a correlation between hepcidin levels and the risk of developing subacute and chronic autoimmune thyroiditis. A reduction in hepcidin levels has been observed in patients with Hashimoto’s thyroiditis in whom euthyroidism has been reverted [46]. Thus, it is possible that hypothyroidism results in increased hepcidin with a consequent reduction in iron absorption. Testosterone is negatively associated with serum ferritin. In obese hypogonadal elderly men receiving testosterone replacement therapy, serum ferritin levels significantly decreased, suggesting a regulatory function of testosterone on ferritin synthesis. Indeed, in obese patients, decreased testosterone levels can lead to an increase in serum ferritin [47,48]. Insulin resistance and metabolic syndrome also appear to positively correlate with serum ferritin levels [49]. Studies conducted in pregnant women did not consider the role of human chorionic gonadotropin (hCG) on TSH levels. In fact, hCG has structural similarities with TSH, allowing it to bind to TSH receptors at the thyroid level, stimulating the secretory activity of the gland. This explains the slight increase in serum free thyroxine levels during the first weeks of pregnancy, accompanied by a reduction in serum TSH concentration [50]. Finally, the different studies considered different SF cut-offs for the diagnosis of ID. However, as indicated in the guidelines, all studies report SF values <20 ng/dL as the cut-off for the diagnosis of ID, and the lower limit of normal for most laboratories is between 15 and 30 ng/dL [51].

This systematic review and meta-analysis has some limitations. First, the lack of studies conducted on the general population. Most of the studies are performed on pregnant women and this could affect the results. Second, the reviewed studies considered different SF cut-offs for the diagnosis of ID. Furthermore, most studies in the literature that include a control group are cross-sectional, so we do not know the results of prospective or longitudinal studies. Finally, many of the studies analyzed do not consider the iodine status of the patients examined, which as is known influences thyroid function. In addition to iodine, other parameters such as inflammation and the action of other hormones affect iron status and thyroid function and could therefore influence the results.

In conclusion, ID may adversely affect thyroid function and autoimmunity, especially in some groups, such as pregnant women and women of childbearing age. The articles analyzed in this systematic review and meta-analysis are cross-sectional studies, and it was not possible to distinguish randomness between ID and thyroid function. The relationship between trace elements and thyroid disorders is still unclear, and more research is needed to clarify this issue and improve our understanding of how trace elements mediate thyroid function and metabolism. Prospective randomized controlled trials are needed to clarify the importance of iron’s nutritional status on thyroid health, including in the general population or in other patient groups.

## Figures and Tables

**Table 1:** Inclusion and exclusion criteria of the current systematic review, according to the PECOS model [17].

	Inclusion	Exclusion
Population	Adults, pregnant women	Patients with comorbidities or taking drugs that affect thyroid function or iron status, age < 18 years.
Exposure	Iron deficiency	-
Comparison	Subjects without ID	-
Outcome	TSH, FT4, FT3, TPOAb, gAb	-
Study Type	Cross-sectional study, randomized controlled study, case–control, cohort	*In vitro*, animal studies, case reports, editorials, communications, reviews, and meta-analyses

Abbreviations. FT3, free triiodothyronine; FT4, free thyroxine; ID, iron deficiency; SF, serum ferritin; TgAb, antithyroglobulin antibodies; TPOAb, anti-thyroid peroxidase antibodies; TSH, thyroid-stimulating hormone.

**Table 2:** Main characteristics of the studies included in the analysis.

	Type of Study	Age (y)	Type of Population	Population (*n*)		Diagnostic Criteria for ID (SF)	Items and Reference Range	TSH		FT4		FT3		TPOAb		TgAb	
				ID	Controls												
								ID	Controls	ID	Controls	ID	Controls	ID	Controls	ID	Controls
Wang et al., 2022 [20]	CSS	29 (25–33)	Chinese Pregnant women	387	1831	<20 ng/dL	TSH (0.24–4.2 mIU/L), FT4 (12–22 pmol/L), FT3 (3.1–6.8 pmol/L)	1.54 (0.92–2.35)	0.93 (0.43–1.56)	13.23 (11.26–16.08)	16.58 (14.67–18.67)	4.15 (3.51–5.04)	4.79 (4.33–5.32)	NR	NR	NR	NR
Okuroglu et al., 2020 [21]	CSS	31.5 ± 8.4	Non-pregnant women of reproductive ages	203	155	<15 ng/dL	TSH (0.35–4.2 mIU/L), FT4 (0.58–1.64 ng/dL), FT3 (1.71–3.71 pg/mL), AbTPO (<5.6 IU/mL), AbTg (<10 IU/mL)	1.90 ± 0.97	1.74 ± 0.91	0.96 ± 0.11	0.97 ± 0.11	2.77 ± 0.42	2.81 ± 0.60	8.37%	3.87%	9.85%	7.10%
Zhang et al., 2020 [22]	CSS	28.3 ± 0.1	Chinese pregnant women	373	1219	<20 ng/dL	TSH (0.27–4.2 mIU/L), FT4 (12–22 pmol/L), AbTPO (<34 IU/mL), AbTg (<115 IU/mL)	1.85 (0.01–7.84)	1.69 (0.01–10.2)	13.94 (8.91–29.82)	14.63 (8.22–47.24)	NR	NR	3.8%	5.2%	4.6%	3.1%
Zhang et al., 2019 [23]	CSS	28.3 ± 3.6	Chinese pregnant and non-pregnant women	638	8229	<15 ng/dL	TSH (0.14–4.87 mIU/L), FT4 (NR), AbTPO (<34 IUmL), AbTg (<115 IU/mL)	2.06 (1.17–3.09)	1.99 (1.14–3.05)	14.88 ± 2.32	16.05 ± 3.19	NR	NR	8.9 (5–15.6)	8.21 (5–13.82)	12.33 (10–42.45)	11.35 (10–24.34)
Maldonado-Araque et al., 2018 [29]	CSS	50 ± 17.1	Spanish adult non-pregnant population	523	3323	<15 ng/dL	TSH (µUI/mL), FT4 (pmol/L), FT3 (pmol/L)	1.98 ± 0.07	2.04 ± 0.04	14.83 ± 0.10	15.16 ± 0.07	4.91 ± 0.03	5.01 ± 0.02	NR	NR	NR	NR
He et al., 2018 [24]	CSS	27.2 ± 4.5	Pregnant women	69	140	<12 ng/dL	TSH (0.27–4.2 mIU/L), FT4 (12–22 pmol/L), AbTPO (<34 IU/mL), AbTg (<115 IU/mL)	3.25 ± 1.72	2.36 ± 1.48	14.26 ± 1.45	14.45 ± 1.54	NR	NR	9.7 (9.3–14.3)	10.4 (9.6–14.5)	10 (10–15.95)	10.02 (9.2–15.09)
Fu et al., 2017 [25]	CSS	27 ± 3	Chinese pregnant women	932	832	<20 ng/dL	TSH (0.98–2.26 mIU/L), FT4 (8.85–12.25 pmol/L), FT3 (4.06–5.06 pmol/L)	1.6 (1–2.3)	1.4 (0.7–2.2)	10.36 (8.8–12.2)	10.88 (8.9–13.44)	4.4 (3.9–5)	4.6 (4.2–5.2)	NR	NR	NR	NR
Li et al., 2016 [26]	CSS	28.5 ± 3.7	Pregnant women	431	2150	<20 ng/dL	TSH (0.1–2.5 mIU/L), FT4 (11.5–18.8 pmol/L), AbTPO (<5.6 mIU/mL)	1.35 (0.71–2.4)	1.07 (0.6–1.67)	13.22 (11.95–14.84)	13.99 (13.12–15.07)	NR	NR	3 (23.6%) (3–4.28)	3 (11.2%) (3–3)	NR	NR
Veltri et al., 2016 [27]	CSS	30 (15–47)	Pregnant women	674	1226	<15 ng/dL	TSH (0.3–4 mIU/L), FT4 (0.8–2 ng/dL), AbTPO (<60 kIU/L)	1.5 (0.0–9.6)	1.3 (0.0–30.5)	1.0 (0.7–2.2)	1.1 (0.6–3.1)	NR	NR	29 (10%) (15–9704)	28 (6%) (25–13,000)	NR	NR
Yu et al., 2015 [28]	CSS	28.9 ± 3.6	Chinese pregnant and non-pregnant women	143	4249	<12 ng/dL	TSH (0.14–4.87 mIU/L), FT4 (12.4–20.7 pmol/L)	1.94 (1.2–2.73)	1.92 (1.12–2.85)	15.22 ± 2.61	16.06 ± 2.47	NR	NR	NR	NR	NR	NR

Abbreviations: AbTg, antithyroglobulin antibodies; AbTPO, anti-thyroid peroxidase antibodies; CSS, cross-sectional study; FT4, free thyroxine; FT3, free triiodothyronine; ID, iron deficiency; SF, serum ferritin; TSH: thyroid-stimulating hormone.

**Table 3:** Quality of evidence assessment of the included studies (results of the Cambridge Quality Checklist [19]).

Study Name	Type of Study	Cambridge Quality Checklists		
		Checklist for Correlates	Checklist for Risk Factors	Checklist for Causal Risk Factors
Wang et al., 2022 [20]	CSS	4	1	5
Okuroglu et al., 2020 [21]	CSS	3	1	5
Zhang et al., 2020 [22]	CSS	4	1	5
Zhang et al., 2019 [23]	CSS	5	1	5
Maldonado-Araque et al., 2018 [29]	CSS	5	1	5
He et al., 2018 [24]	CSS	4	1	5
Fu et al., 2017 [25]	CSS	5	1	5
Li et al., 2016 [26]	CSS	5	1	5
Veltri et al., 2016 [27]	CSS	5	1	5
Yu et al., 2015 [28]	CSS	4	1	5

Abbreviations. CSS: cross-sectional study.

## Data Availability

Data are contained within the article.

## Supplementary Materials

The following supporting information can be downloaded at: https://www.mdpi.com/article/10.3390/nu15224790/s1, Table S1: MOOSE Checklist for Meta-analyses of Observational Studies; Table S2: PRISMA 2009 Checklist. References [15,52] are cited in supplementary materials.
