# Supplementary material for: Relationship between Iron Deficiency and Thyroid Function: A Systematic Review and Meta-Analysis
Source: Nutrients. 2023 Nov 15;15(22):4790. doi: 10.3390/nu15224790 (PMC10675576; doi:10.3390/nu15224790)
Supplement: Supplementary file 1 [file nutrients-15-04790-s001.zip › nutrients-2672024-Supplementary-Table-S1.pdf]

MOOSE Checklist for Meta-analyses of Observational Studies

Item No	Recommendation	Reported on Page No
Reporting of background should include		
1	Problem definition	2
2	Hypothesis statement	2
3	Description of study outcome(s)	2
4	Type of exposure or intervention used	2
5	Type of study designs used	2
6	Study population	2
Reporting of search strategy should include		
7	Qualifications of searchers (eg, librarians and investigators)	5
8	Search strategy, including time period included in the synthesis and key words	5
9	Effort to include all available studies, including contact with authors	-
10	Databases and registries searched	5
11	Search software used, name and version, including special features used (eg, explosion)	-
12	Use of hand searching (eg, reference lists of obtained articles)	5
13	List of citations located and those excluded, including justification	-
14	Method of addressing articles published in languages other than English	-
15	Method of handling abstracts and unpublished studies	5
16	Description of any contact with authors	-
Reporting of methods should include		
17	Description of relevance or appropriateness of studies assembled for assessing the hypothesis to be tested	5-6
18	Rationale for the selection and coding of data (eg, sound clinical principles or convenience)	5-6
19	Documentation of how data were classified and coded (eg, multiple raters, blinding and interrater reliability)	6
20	Assessment of confounding (eg, comparability of cases and controls in studies where appropriate)	-
21	Assessment of study quality, including blinding of quality assessors, stratification or regression on possible predictors of study results	6-7
22	Assessment of heterogeneity	7
23	Description of statistical methods (eg, complete description of fixed or random effects models, justification of whether the chosen models account for predictors of study results, dose-response models, or cumulative meta-analysis) in sufficient detail to be replicated	7
24	Provision of appropriate tables and graphics	-
Reporting of results should include		
25	Graphic summarizing individual study estimates and overall estimate	Fig 1
26	Table giving descriptive information for each study included	Table 3
27	Results of sensitivity testing (eg, subgroup analysis)	Supplementary Figure 1 – Supplementary Figure 3 – Supplementary Figure 5

28	Indication of statistical uncertainty of findings	-

Item No	Recommendation	Reported on Page No
Reporting of discussion should include		
29	Quantitative assessment of bias (eg, publication bias)	10-13-16
30	Justification for exclusion (eg, exclusion of non-English language citations)	7
31	Assessment of quality of included studies	Table 2
Reporting of conclusions should include		
32	Consideration of alternative explanations for observed results	19-22
33	Generalization of the conclusions (ie, appropriate for the data presented and within the domain of the literature review)	19-22
34	Guidelines for future research	-
35	Disclosure of funding source	-

From: Stroup DF, Berlin JA, Morton SC, et al, for the Meta-analysis Of Observational Studies in Epidemiology (MOOSE) Group. Meta-analysis of Observational Studies in Epidemiology. A Proposal for Reporting. *JAMA*. 2000;283(15):2008-2012. doi: 10.1001/jama.283.15.2008.
